# Why do GPs hesitate to refer diabetes patients to a self-management education program: a qualitative study

**DOI:** 10.1186/1471-2296-12-94

**Published:** 2011-09-08

**Authors:** Patricia Sunaert, Marie Vandekerckhove, Hilde Bastiaens, Luc Feyen, Piet Vanden Bussche, Jan De Maeseneer, An De Sutter, Sara Willems

**Affiliations:** 1 Department of General Practice and Primary Health Care, Ghent University, Belgium; 2 Department of Clinical & Lifespan Psychology & Experimental and Applied Psychology, Brussels University, Belgium; 3 Department of General Practice, Interdisciplinary Healthcare and Geriatrics, Antwerp University, Belgium

## Abstract

**Background:**

Self-management support is seen as a cornerstone of good diabetes care and many countries are currently engaged in initiatives to integrate self-management support in primary care. Concerning the organisation of these programs, evidence is growing that engagement of health care professionals, in particular of GPs, is critical for successful application. This paper reports on a study exploring why a substantial number of GPs was (initially) reluctant to refer patients to a self-management education program in Belgium.

**Methods:**

Qualitative analysis of semi-structured face-to-face interviews with a purposive sample of 20 GPs who were not regular users of the service. The Greenhalgh diffusion of innovation framework was used as background and organising framework.

**Results:**

Several barriers, linked to different components of the Greenhalgh model, emerged from the interview data. One of the most striking ones was the limited readiness for innovation among GPs. Feelings of fear of further fragmentation of diabetes care and frustration and insecurity regarding their own role in diabetes care prevented them from engaging in the innovation process. GPs needed time to be reassured that the program respects their role and has an added value to usual care. Once GPs considered referring patients, it was not clear enough which of their patients would benefit from the program. Some GPs expressed the need for training in motivational skills, so that they could better motivate their patients to participate. A practical but often mentioned barrier was the distance to the centre where the program was delivered. Further, uncertainty about continuity interfered with the uptake of the offer.

**Conclusions:**

The study results contribute to a better understanding of the reasons why GPs hesitate to refer patients to a self-management education program. First of all, the role of GPs and other health care providers in diabetes care needs to be clarified before introducing new functions. Feelings of security and a basic trust of providers in the health system are a prerequisite for participation in care innovation. Moreover, some important lessons regarding the implementation of an education program in primary care have been learned from the study.

## Background

Within the health care field, there is an increasing awareness of the importance of self-management within the treatment and follow-up of persons with a chronic condition [1]. Patients are handling (self-managing) their condition on a daily basis and the way they do this has a major influence on the outcome of their illness. Most individuals will need support in order to acquire the necessary knowledge, skills and confidence to cope with this task. This is especially the case for diabetes patients for whom self-management support is seen as a cornerstone of good diabetes care. As a result, many countries are currently engaged in initiatives to integrate self-management support for diabetes patients in primary care [2].

Concerning the organisation of self-management education programs, evidence is growing that the engagement of health care professionals is critical for successful application of these programs. In the past, the lack of integration in primary care has resulted in recruitment problems, especially of patients from ethnic minority groups [3]. Further, poor integration may lead to missed opportunities for follow-up and behaviour reinforcement [4]. In particular general practitioners (GPs) are in a position to target interventions to individual patients because of their knowledge of patient's context, biography and illness trajectory. For this reason, recruitment directly from within primary care is seen as a way to enhance patient participation in self-management programs [5].

In Belgium, an education program for type 2 diabetes patients was piloted in primary care during a four-year action research project (2003-2007). The education program was launched as a new service in the region in October 2004 and was part of a complex intervention based on the Chronic Care Model (CCM) [6]. The content of the education program was theory driven (empowerment theory) and tailored to the individual needs of the patient [7,8]. An important role was assigned to GPs in identifying and motivating patients eligible for self-management support. In order to achieve maximum integration within usual care, patients could only enter the program on referral by their GP. An implementation strategy was developed based on the evidence on successful implementation of care innovation [9].

As part of an overall evaluation, the participation rate of patients in the education program and the referral rate by individual GPs were monitored. Participation of patients increased gradually and the adoption of the new service by GPs showed a curve similar as the one described by Rogers [10]. Two years after the introduction of the program in the region, about 70% of all GPs in the region had at least one patient in the program. However, there was a substantial variation in the number of participating patients per GP (1 to 25) and about one-third of all GPs still had no patients in the program. GPs that used the service on a regular basis, experienced the program as supportive and complementary to their own work. In particular, the added value for the participating patients and its impact on daily diabetes care was emphasised [8].

This paper reports on a study exploring why a substantial part of GPs was (initially) reluctant to refer patients to an education program. The main purpose of the study was to explore the GP-perceived barriers regarding the referral to an education program for diabetes patients so that self-management support initiatives could be refined in the future.

## Methods

### Design

Qualitative analysis of semi-structured face-to-face interviews with a purposive sample of GPs who were not regular users of the service.

### Setting

The self-management education program was launched in a well-defined area with 76,826 inhabitants and an estimated number of 2,300 type 2 diabetes patients. In 2004, the region counted 83 GPs (GP to population ratio of 1:925) of whom 70% worked in a single-handed practice. Two trained diabetes educators were appointed to deliver the program in the region. The target population were people with type 2 diabetes on lifestyle treatment and/or oral medication with the focus on newly diagnosed patients, patients with complex problems and patients not reaching the targets on maximal oral therapy.

### Participants

A purposive sample of 20 GPs was selected in order to obtain maximal diversity (degree of adoption, gender, age, practice type). The degree of adoption of the service was defined by the number of patients participating in the program. According to the number of patients entering the program, GP's were assigned to 4 groups: ≥ 5 patients (group 1), < 5 and ≥ 1 patients (group 2), 1 patient (group *3) *and no patient in the program (group 4) (table 1). GPs in group 1 were considered as regular users of the service. GPs from group 2, 3 and 4 were eligible for our study. Of the 20 invited GPs five could not be interviewed: four GPs refused to participate (2 because of time-restraints (group 3), two without a formal reason (group 4)) and one GP was abroad (group 2) when the interviews were planned. Those five GPs were replaced by GPs with the same referral profile and comparable demographic and practice characteristics.

**Table 1:** Characteristics of GPs in relation to referral rate

	Group 1 (n = 16) *≥ 5 patients*	Group 2 (n = 21) *< 5 and ≥ 1 patients*	Group 3 (n = 16) *1 patient*	Group 4 (n = 27) *no patient*	Total(n = 80)
Participant characteristics					

• Female (%)	25	5	19	22	18

• Age in 2007 (mean; years)	48,1	46,1	48,1	48,6	47,8

*Practice characteristics*					

• Single-handed practice (%)	50	62	94	85	74

• Staff support in practice (%)	25	31	21	35	*29*

• ≥ 30 diabetes patients (%)	69	67	77	59	*67*

• Diabetesregister (%)	33	31	15	20	*25*

*Participation in complex intervention *					

• Protocol development (%)	31	5	0	0	*7*

• Attending ≥ 1 training session (%)	94	48	50	33	53

• Participation in audit (%)	63	29	6	7	24

• Referral for insulin initiation (%)	69	48	31	0	33

### Data collection instruments

A flexible topic guide with a loose structure of open-ended questions was used in order to explore experiences and attitudes regarding the service in general, reasons for limited or non-referral to the program and suggestions for adaptation of the program in the future.

### Procedure

First, potential participants were informed about the study by e-mail. Next, the interviewer contacted them by phone and the purpose of the interview was briefly explained. If the GP consented in the interview, an appointment was made. All the interviews were conducted by the second author (MV), a psychologist trained in qualitative interviewing methods. To encourage candid responses we chose for an interviewer not involved in the pilot study. All interviews were audio taped and transcribed verbatim for coding an analysing. The interviews took place in the GP's practices and were conducted from February 2007 through April 2007.

### Ethics

The study was approved by the Ethical Committee of the University of Ghent (Approval number 2004/253) and the Ethical Committee of the University of Antwerp (Approval number 12/07/2004).

### Analysis

Two researchers (MV, PS) were involved in coding the data. The interview data were analysed by the framework approach for policy relevant qualitative research (familiarisation, identifying a thematic framework, indexing, charting, mapping and interpretation) [11]. First, the interview data were analysed in order to identify the main reasons for limited referral of patients to an education program as experienced by the individual GPs. This analysis produced a list of major themes related to the limited uptake of the service. Next, the text material was analysed in order to develop a deeper understanding of the reticent behaviour of the GPs regarding the offer in the region. In this stage the Greenhalgh diffusion of innovation framework, a theoretical model of complex change, was used as background and organising framework [12]. The Greenhalgh model is based on a systematic review of the literature regarding the diffusion of innovations in health care organisations addressing the question: 'How can we spread and sustain innovations in health service delivery and organisations?' and identifies key domains in which factors influencing uptake and implementation are found. In this study the innovation concerned was the education program, the organisation where the innovation was intended to spread was the network of GPs in a defined region. The same analysis procedure has been used in both steps. First, two researchers performed the coding independently (MV and PS). Next, consensus was reached on the aggregated data through discussion (researcher comparison).

## Results

### Participant characteristics

Table 2 presents participant characteristics.

**Table 2:** Characteristics of participating GPs

	GROUP 2 (n = 5) *< 5 and ≥ 1 patients*	GROUP 3 (n = 5) *1 patient*	GROUP 4 (n = 10) *no patient*	TOTAL (n = 20)
Age in 2007(mean; years)	51.8 (46-57)	43.8 (33-52)	46.6 (31-63)	47,2

Female (number)	/	2	2	4

Single-handed (number)	4	4	9	17

### Reasons for non or limited referral

GPs expressed several reasons for non or limited referral of patients to the education program. Through coding and analysing, a number of key themes linked to different phases in the adoption process emerged from the interview data [13]. Most GPs mentioned more than one reason. Further, some GPs (not all) evolved in the adoption process during the intervention period. As a result, reasons linked to different phases of the change (adoption) process can be seen for one and the same GP. Once GPs moved ahead in the adoption process, other barriers could turn up. The results are presented in relation to the components of the Greenhalgh model (table 3) and the strategies we have used to enhance implementation of the program in the region [14]. The most important reasons are discussed and illustrated with some quotes.

**Table 3:** Key themes in relation to the components of the Greenhalgh model

Components of the diffusion of innovation model	Strategies used to enhance implementation	Key themes
1. Attributes of the innovation To be successfully and widely adapted, an innovation must be seen by potential adopters as having: • Relative advantage • Simplicity • Compatibility with existing values and ways of working • Trialability • Observability • Potential for reinvention	• theory-driven program based on current evidence • well-trained diabetes educators • integrated in primary care • no financial threshold for patients • possibility of home visit	• extra administrative workload • motivation of patients requires extra consultation time • doubts about the added value of the service • potential benefits not visible enough • distance to the centre

2. Concerns of potential adopters Adoption is a process, not a one-off event, and is influenced by concerns, including: • Prior to adoption (what are its properties and potential benefits? What will it cost me?) • During early use (how do I make it work?; when and how should I use it?) • During established use (how can I alter or improve it?)	• interdisciplinary care protocol with clear job descriptions • strengthening of GPs' role in diabetes care • referral by GP obliged • advice regarding the target groups	• fear of further fragmentation of diabetes care • fear of negative interference with the doctor-patient relationship • fear of losing control over therapy • uncertainty about job boundaries • doubts about which of their patients will benefit (the most) • lack of motivational skills • patients are not asking for the service themselves

3. Communication and influence An individual's decision to adopt an innovation is influenced by: • Mass media • Interpersonal influence	• information campaign targeting GPs, patients and other health care providers	• confusion regarding the aims of the project • limited awareness of the program among patients • negative attitude of peers towards the program

4. Organisational antecedents for innovation Organisations may be more or less innovative. Differences are explained by several factors: • Absorptive capacity for new knowledge • Leadership and management • Risk-taking climate • Effective data capture systems • Slack resources	• establishment of a local steering group • appointment of a program manager • involvement of regional stakeholders • sufficient financial resources (for the course of the pilot)	• no tradition with the initiation of care innovation in primary care

5. Organisational readiness for innovation Readiness includes: • Innovation-system fit • Tension for change • Balance between supporters and opponents • Specific preparedness	• survey among health care providers at the start of the project, exploring the needs regarding diabetes care in the region	• non-referral as a way to express dissatisfaction with their current role in the health care system • disbelief that project results can influence health policy

6. The implementation process Implementing a complex innovation, and making sure it becomes business as usual, is a highly non-linear process, typically characterised by shocks and setbacks. Critical success factors include: • Appropriate of change model • Good project management • Human resource issues • Alignment between new and old routines	• establishment of a local steering group • establishment of study groups • appointment of a program manager • balanced implementation plan ('help it happen' strategy)	• tend to forget about the service

7. Linkage Innovation is more likely when there is: • Early and ongoing dialogue between developers of the innovation, the change agents charged with promoting its adoption, and its users • Communication within the organisation and between similar organisations	• involvement of GPs in program development, initial via their QPRGs, later on via the study groups • involvement of all health care providers involved in diabetes care via study groups	• not used to being involved in care innovation development

8. The broader context Innovation in organisations is more likely to be successful when there is a 'following policy wind', a conductive socio-political climate, and specific incentives and mandates at national level	• integration of the program in primary care • financial resources provided by project funding • regular contact with the commissioners of the study	• feelings of frustration and insecurity regarding GPs' position and role in health care • disbelief that project results can influence health policy • uncertainty about continuity of the program

#### Attributes of the education program

Relative advantage is a sine qua non for adoption of an innovation. In this case, the cost-benefit analysis of the program remained negative for a substantial number of GPs. Several GPs reported that the program increased workload instead of supporting them in patient care. In fact, completing the administrative procedures (informed consent, referral letter) and motivating patients to enter the program required extra consultation time.

"We need time to motivate the patient to enter the program. We also need sufficient time to listen to the patients' problems. This is conflicting. Moreover, so many other projects are also claiming our attention (screening for breast cancer, screening for colon cancer, ..). In the end there is no time left for the real job."(4, 2)

"The administrative procedures are a major barrier. They have a negative impact on the regular doctor-patient relationship. My patients do not understand the reason for this paperwork. When they need a nurse in usual care, they do not have to sign papers either."(3,2)

The perception of extra administrative workload was unfortunately increased by another component of the complex intervention in the region. Concurrently with the launch of the education program GPs were invited to participate in an audit of diabetes care in the region. As a result some GPs thought they had to participate in the audit in order to be allowed to refer patients to the centre. This has been an important disturbing factor, especially at the start of the program.

"It doesn't feel like the project is supporting my work as a GP. Until now, I have not experienced any positive effect of the project. On the contrary, the project is more of a waste of valuable time to the GP. We already take care of our patients; the registration of quality data has no added value."(4, 2)

Furthermore, the potential benefits of the education program remained too vague. Once diagnosed, diabetes patients are expected to take part in managing their symptoms, treating their condition, coping with physical and psychosocial consequences and in making lifestyle changes. Almost all GPs mentioned that (some of) their diabetes patients struggled with the uptake of the daily care of their illness and could benefit from extra support. However, some of them were not convinced that the program was an appropriate answer to this problem or doubted whether the program would result in better outcomes.

"Many of my patients will rather take an extra pill than change their lifestyle. However, I do not know if there is a solution to this problem. If they don't listen to their GP, will they listen to another person? Some people are difficult to motivate. I do not tend to refer these patients to the program because I assume that they will not be open for it."(4,8)

"I appreciate the launching of an education program in the region. This initiative will benefit our patients. As we are limited in our possibilities to educate patients ourselves, the program is in a way also supporting us. We can save time. However, I am not sure if this initiative will result in better outcomes. Probably it will be difficult to evaluate."(2,1)

This perception often changed once GPs had a patient in the program. Due to the positive experiences among their patients, the potential benefits of the program became more apparent, e.g. GPs experienced that their patients were more receptive for advice. As a result, these GPs intended to use the program in the future.

"At the start I refused to cooperate with the project and to refer patients to the centre. My perception of the project was negative mostly because of the extra administrative workload. With time my attitude towards the project changed in a positive way. I now realise that the program works very well and that it supports our patients. Therefore I'm referring more and more patients to the centre."(2, 3)

"At the beginning I experienced the program as too vague. However, once you have read the information and you have a patient in the program, the purpose becomes more evident. In my opinion it is a good program. But you need to take the first step."(3,5)

As the service was not available in the past, some GPs have invested time in patient education themselves. These GPs were not convinced of the added value of an educator in diabetes care. They experienced no problems managing diabetes in practice and argued that patients probably preferred to receive information from them and not from an unfamiliar person in a centre, who does not know the context of the patient.

"Perhaps some GPs want to delegate this part of diabetes care (education) to another care provider. I prefer to inform my patients myself because I think I can offer more continuity than a supplementary program. Therefore I'm willing to put a lot of energy in this aspect of care. My patients can always contact me when they have questions. "(4,7)

Others have organised their own network for education. In some cases, the educator was seen as an intruder in established relationships with other care providers.

"I cooperate very well with other care providers (nurse, dietician) in my neighbourhood. I do not feel the need to use the program because one of the nurses I work with is qualified to give education. I have no reason to refer my patients to the centre."(4,4)

"The introduction of a new function competes with the already available workforce in the region (nurses of different organisations). The introduction of an educator in primary care disturbs our relationship with the nurses in the field. The nurses I am used to working with know the patient and the patient's context very well."(4,3)

A practical barrier often mentioned by the GPs was the distance to the centre, located centrally in the city. According to the GPs, their patients were often elderly and less mobile people for whom the distance to the centre was an extra barrier to enter the program. The program targeted patients from a defined geographical area, a small city (40.000 inhabitants) surrounded by eight communities. The distance barrier was mainly referred to by GPs working in the surrounding communities. To overcome this barrier, the possibility of home visits was introduced, but some GPs mentioned they had forgotten about this option.

"The accessibility of the program is an obstacle to some of my older patients. The distance from their homes to the centre is too far and therefore they need to ask their family for transport."(3, 5)

"The program should be organised as near to the patient as possible. The possibility of home visits should be expanded and perhaps one should consider organising the program in the GP's practices. The threshold for participation has to be held as low as possible."(4,2)

#### Concerns of potential adopters (GPs)

Although a clear job description was provided for the educators, most GPs experienced the introduction of a new function (educator) in the region as a potential threat. GPs were afraid to lose their patients (once more), this time not to an expert in secondary care (the specialist) but to an expert in primary care (educator). They feared further fragmentation of diabetes care and stated that the introduction of a new function would undermine the doctor-patient relationship.

"My role in diabetes care is limited. There are already two diabetes centres (hospital) in the region and now another centre in primary care is limiting the role of GPs in diabetes care. For this reason I decided not to participate. Moreover, the referral of a patient to a centre often means the end of the established doctor-patient relationship. Diabetes-related consultations decrease; you are not the only expert anymore."(4, 5)

"The introduction of a new function (educator) in primary care limits the role of the GP to the referral of the patient. This project is once more an illustration of the ongoing fragmentation of care (COPD, asthma, ..)."(4,3)

For some GPs, job boundaries were not always clear enough from the start, e.g. is an educator allowed to change therapy? They were afraid to lose control over therapy and to be left with the paperwork. Some GPs emphasised the need for more communication regarding collaboration modalities.

"My objection against the program is that the content of the program overlaps with our job. When it comes to dietary advice, the project can probably do a good job, as well as for lifestyle advice in general. But I do not agree with a centre giving therapeutic advice regarding glycaemic control. I like to have control, and I cannot accept that the centre takes over my role. There should be more emphasis on clear job descriptions."(3, 1)

Again, these concerns usually diminished once GPs had a patient in the program and perceived that the educator respected the task agreements.

"At the start I was afraid that the centre would take diabetes care away from primary care. Later on I became convinced of the added value of collaborating with the program. I appreciate their way of working. The communication with the centre is very good, especially via e-mail." (2,5)

Another point of concern was the selection of patients for the program. To most GPs, once they considered using the program, it was not clear enough which of their patients would benefit (the most) from education. As a result the selection criteria varied widely. Some GPs proposed the program to all of their patients, others were more restrictive and e.g. proposed the offer only to their motivated patients.

"A number of patients in my practice did not qualify for the program. In many cases current treatment is successful, so I see no reason to refer them to the program. In other cases, patients have too many problems, so I prefer referring them to the specialist. Some other patients were too old for the program (e.g. living in a home for the elderly)."(3,4)

Further, some of the GPs experienced they lacked the skills to motivate patients for this kind of service, which might explain why many of their patients preferred not to participate. Ultimately, it is the patient who decides whether he enters the program or not. The training sessions mainly focussed on the content of the program and less on the skills needed to motivate patients for the program.

"It is a positive initiative but in my experience it is not easy to motivate patients to enter the program. Maybe I lack motivational skills."(3,4)

The efforts made to motivate patients varied widely. Some GPs displayed a poster in their waiting room, others distributed leaflets and some invested extra consultation time. The fact that, in spite of the efforts made to inform patients regarding the education offer (mass media, leaflets, poster campaign), most patients did not ask themselves to enter the program, made the job harder.

"Until now, none of my patients has volunteered for the program. If my patients had expressed the need for it, I would certainly have referred them to the centre. However the patients are not yet motivated to enter this kind of program. We have to push them." (4,7)

#### Communication and influence

GPs were first informed about the program via their local quality peer review groups (QPRGs). These groups are part of a quality assurance program for GPs and specialists in Belgium since 1996 and meet at least four times a year. The QPRGs turned out to be an effective channel for information transfer. Almost all GPs heard about the program through their QPRG. Later on information was spread on a regular basis through different channels: mass media, e-mail, training sessions, and interpersonal contacts. Most GPs were positive about the information campaign, and the QPRGs and training sessions were considered as the most effective information channels. Written documents and e-mails were often ignored.

"We were well informed about the program; we were invited to visit the centre and training sessions were organised."(4,8)

Nevertheless, a group of GPs experienced the information campaign at the start as confusing (see linkage) and some GPs advised more aggressive information methods, comparable with those used by pharmaceutical companies (see implementation process).

"The information campaign was rather chaotic at the start. I had the impression that the information campaign was not well-prepared. It was not clear enough what we could expect from the program and how we could use it. Later on, we received a lot of written information and once I had a patient in the program, everything became more evident."(3,5)

In the course of the interviews, several GPs referred to the negative attitude towards the program (and the global project) among their peers to reinforce their own concerns. Most concerns, expressed by their peers were linked to the fear of further fragmentation of care, fear of losing the patient (once more) and disbelief in the aims of the project.

"Many of my colleagues are reluctant to cooperate with the program. They experience the project as a way for the government to demonstrate that GPs are not performing well in diabetes care. The project is not aimed at supporting primary care, and it comes across as criticism on the quality of diabetes treatment in primary care. What most GPs fear is to lose another part of the job."(2,4)

#### Organisational antecedents and readiness for innovation

In the Belgian context, GPs have little or no tradition with the initiation of care innovation initiatives by themselves. Further, involvement of primary care in policy decision making is limited. As a result, GPs are mostly receptive receivers of innovations planned by other partners in health care (government, hospital, care provider organisations). This is probably an important reason why adoption of innovations among GPs is often moderate to poor (at the start). In this case, we tried to overcome this barrier by involving GPs (and other care providers) in the development of the program. This strategy was enabled by the presence of regional GP networks and the appointment of a program manager in the region. First, cooperation was obtained from the chairman of the regional GP organisation. Next, individual GPs were reached through their QPRG. However, the strategy to involve individual GPs in the development of the program was less successful (see linkage).

"We were involved in the project from the beginning. The starting up was totally different from all the other projects that have been introduced recently (e.g. clinic for obesity). Most of the time the hospitals are taking the leading and GPs are barely involved. I recognise, it is not easy to involve GPs in the development process of all these projects. A delegate needs to be assigned for each project and GPs do not always have the time or interest. Furthermore, most of this work is done for free."(3,4)

In the course of the interviews, many GPs expressed their dissatisfaction with their current role in the health care system (see broader context). Especially the relationship with secondary care was a point of concern for most GPs. Most GPs stated that they have progressively lost territory to specialist care. Accordingly, several GPs were suspicious regarding the new service in the region, fearing that this initiative would further undermine their position in health care. Although the aim of the project was to explore how primary care could be strengthened in chronic care delivery, some GPs experienced the project as another strategy to shift diabetes care towards secondary care, as a method to demonstrate that GPs are not performing well. As a result, for a substantial number of GPs, non-referral was a way to protest against (their perception of) the current evolutions in the organisation of the health care system. They did not believe that the project would lead to a fundamental change.

"I regret that diabetes care is fragmented once more, now in primary care, between GPs and the centre. I do not agree with this evolution and for this reason I did not refer any patient, I am consequent. The project aims to restore the central position of the GP but I do not experience it this way. The decision to establish such a centre bothers me, not the people who work there (I think they do a good job)." (4,6)

#### The implementation process

Until recently, most care innovations in the Belgian context were hardly accompanied by an implementation plan. This is probably another reason why adoption of innovations among GPs is often moderate to poor (at the start). In this case, we tried to overcome this barrier. An implementation plan was developed (information campaign, training sessions, personal contact, website, e-mail, ..) and supervised by the program manager (in collaboration with the research team). The implementation plan relied on the 'Help it happen' strategy. Some GPs started to use the service, but stopped later on. Once the information campaign weakened, they forgot to mention the program to their patients. These GPs advised to use a more intensive promotion campaign with frequent interpersonal contact in order to enhance and maintain participation.

"At the beginning I referred some patients to the centre. But after a while, one forgets to propose the opportunity to the patients. There were some problems with the implementation of the program and follow-up has to be more active. We need an approach comparable to the approach used by the pharmaceutical companies. Currently only those GPs who are really interested remain in the project."(2,1)

#### Linkage

In order to achieve intervention-adopter fit provisional project plans were proposed to GPs (via their QPRGs) with the appeal to bring in their vision regarding high quality diabetes care. This strategy was not a success in the current context. Several GPs perceived this approach as a lack of professionalism and preparation of the research team. They expressed the need for concrete, relevant information and were reluctant to participate in the development of a program. Their advice was to introduce a clear concept with the possibility of adaptations in the future.

"I had the impression that the project team was still reflecting on the content of the project. They need to do their homework instead of debating with the target group about the modalities of the project. It is not our job to conceive a project. They should introduce a clear concept and provide the possibility to make adaptations later on."(2,1)

Consequently, the involvement of GPs in program development was organised through participation of interested GPs in study groups.

#### The broader context

The education program was launched in a context where some of the preconditions to facilitate chronic care delivery in primary care (patient list, gatekeeper function, staff support, IT support) are still not or only moderately fulfilled. Furthermore, initiatives to support diabetes care were until recently mainly hospital-based, leading to a transfer of patients to secondary care. This has led to frustration among GPs who feel powerless as they cannot 'compete' with the expertise and service delivered in secondary care. Several GPs expressed their dissatisfaction about their current role in (diabetes) health care.

"Our attitude regarding the project was probably influenced by former experiences. I have been doing this job for 20 years now. We've gradually lost territory to the specialists, e.g. patients on insulin therapy could suddenly get all their material for free in diabetes centres. This way, we lose patients. What remains is a letter from the specialist every two months and a thick patient file."(2,3)

"They wanted to compare the quality reached in primary care with the quality of care in the diabetes centres. This is not a correct starting point. There will be an enormous bias; hospital care is currently better organised and supported. We need to motivate our patients to consult the centre, to consult the ophthalmologist, ... In the hospital all disciplines are available in one location. We cannot compete with hospitals. It is obvious that the result in the hospital will be better."(3,5)

Although on the one hand efforts were made in the project to strengthen primary care at the regional level (steering group, program manager, study groups) and on the other hand, to strengthen the role of GPs in diabetes care (interdisciplinary care protocol with clear task descriptions and central role for GPs) a number of GPs refused to refer patients for reasons related to the heath care context, even at the end of the study period.

"I do not believe in this project and that's why I did not participate. In the past few years diabetes care has gradually moved from primary to secondary care, mostly under pressure of specialists. As a consequence specialists are currently overwhelmed by primary care work and the cost of diabetes care is rising. Health policy leaders have the intention to turn this trend, but I don't believe specialists really want to return some responsibilities to the GPs. I have the impression that this project will not change health care organisation fundamentally. It's only a small adaptation of the existing system." (4,1)

Linkage with the commissioners of the pilot study was maintained on a regular basis (at least two times a year). Continuation of financing depended on a positive evaluation of the project. Consequently, tension mounted between the positive results longed for by the commissioners and the moderate success of the program in the field. Several GPs mentioned that the program introduced a new way of working and should be given time to grow. Uncertainty about continuity was an additional barrier to take up the service.

"At the start of the program, it wasn't clear if it would be continued after the pilot project. If an offer is temporary, many GPs hesitate to refer patients and to invest much energy in it. This was another threshold to participate. I think it is an offer that needs time to have an effect. First GPs need to be convinced of the program's added value, and then these GPs need to motivate their patients. This takes time, and we don't see our patients each week. "(3,2)

## Discussion

### Summary of the main findings

A range of barriers, related to different components of the Greenhalgh model, emerged from the interview data. One of the most striking ones was the limited readiness for innovation among GPs. Feelings of frustration and insecurity regarding their own role in (diabetes) care prevented them from engaging in the innovation process. They were afraid that the program would lead to further fragmentation of diabetes care and undermine the doctor-patient relationship. Similar concerns were expressed by almost all GPs (even those who became regular users), but part of them overcame these barriers, often mentioning the first patient-related contact with the educator as a reassuring factor. In general, GPs with at least one patient in the program were positive regarding the content of the program and the collaboration with the educator. This, strengthens our findings that regular users are enthusiastic about the program. Once GPs considered using the program, the selection of patients for the program was a point of concern. They were not sure which of their patients would benefit from the program. Further, some GPs expressed the need for training in motivational skills, so that they could better motivate their patients for the service.

### Findings in relation to the literature

Resistance to innovation is a well-known and universal phenomenon. GPs needed time to be reassured that the program respects their role and has an added value to usual care. Similar concerns were registered among GPs regarding the introduction of the Expert Patients Programme (EPP) in the UK [15]. However, one can expect that in a context where GPs feel insecure about their own role in health care it will take more time to build the trust needed to engage in a program. Moreover, these feelings were probably also reinforced by the limited tradition in interdisciplinary collaboration and the current payment system (fee-for-service). A recent publication, evaluating the referral rate of patients to a geriatric day hospital in the Belgian context, reported similar findings [16]. As mentioned in a previous publication, health policy leaders are aware of the fact that the role of GPs needs to be clarified and reinforced but fundamental decisions (patient list, gatekeeper function) fail to occur, partly because of the complexity of the decision process in the Belgian context and the weak power of GPs to influence this process [6]. A review of the Sharing Health Care Initiative (SHCI), a national program (2001-2004) testing different self-management support models in the real world setting of Australia, also reported problems in obtaining referrals from GPs. For this reason some projects changed their recruitment strategies. Projects which had strong relationships with local health services had less problems achieving multidisciplinary cooperation from GPs, community nurses and allied health practitioners. E.g., one project, building on existing relationships with local GPs, was able to recruit clients through a combination of direct referral by GPs and a review of patient lists. The projects, using a resource intensive approach of reviewing patients lists to identify potential clients, appeared most successful in recruiting participants with greater support needs [17]. It is worthwhile to explore this strategy in our context in the future, but first more research is needed to support GPs (or other staff members) in identifying patients who will benefit from a particular education program. Uncertainty about the benefits of the program and limited local evidence on the impact of such programs on patients' self-care abilities, known barriers for engagement by health care providers, were also mentioned by GPs in our study [18]. As recommended by the implementation literature we made efforts to assure that the education program was compatible with the needs of the adopters. Most GPs preferred to be involved once a clear concept was developed, giving them the opportunity to adapt the proposal. This strategy has recently also been reported by some care groups in the Netherlands [19].

### Strengths and limitations of the study

An important strength of this study is that we were able to reach a group which is not often represented in studies, namely GPs who are reluctant to use a new service in a real world context. Where, in the past, non participants were often seen as 'unwilling' individuals, their opinions are currently valued in relation to the evaluation and adaptation of interventions [20]. The results of this study are, for this reason, complementary to the study findings among regular users and are useful to enhance the engagement of GPs in self-management support initiatives in the future. Further, we can assume that the findings reflect the opinion of the target group of the study. In order to encourage candid responses the interviews were conducted by a trained interviewer (MV), who was not involved in the original pilot study. According to MV most GPs were cooperative and willing to reflect on the introduction of the service in the region. Furthermore, the atmosphere during the interviews was mostly open and friendly. Another strength of the study is the use of the Greenhalgh model in the data analysis process. In the past this model has showed to be helpful to reflect on the spread of complex innovations in a specific context [14-21]. Investigating the interview data according to this model was very useful to clarify the problem of limited uptake and to formulate lessons for future self-management support initiatives.

We have to consider some limitations too. In order to categorise the GPs, we defined referral rate by the number of patients entering the program. This is probably an underestimation of the real referral rate. However, according to the interview data, GPs were assigned to the appropriate groups. Further, the education program was part of a complex intervention in the region. Some other components of the intervention may have (positively or negatively) influenced the attitude of GPs regarding the education program. According to the interview data, e.g., the audit and feedback component of the complex intervention increased the perception of administrative workload and was for some GPs an extra barrier to use the program.

## Conclusion

There is growing evidence that engagement of health care professionals, in particular of GPs, is critical for the successful application of self-management support programs. However, the engagement of GPs in the recruitment of patients is not self-evident, even when efforts are made to integrate the program in primary care, as in our case. The results of this study contribute to a better understanding of the reasons why GPs hesitate to refer patients and the strategies needed to enhance their engagement in the future. First of all, the role of GPs in (diabetes) care needs to be clarified before introducing new functions. This can partly be resolved at the regional level, but actions on the national health policy level are necessary too. Feelings of security and a 'basic trust' of providers in the health system are a prerequisite for participation in care innovation. Moreover, some important lessons regarding the implementation of an education program in primary care have been learned from the study:

• Limit the administrative workload.

• Make potential benefits of an education program (more) visible for GPs and patients and give clear advice concerning the group of diabetes patients who will benefit (the most).

• Increase awareness among patients about the availability of an education program.

• Deliver the service by preference in the same setting as the GP, or as close as possible.

• Create opportunities for GPs to train motivational skills.

• Use the first contact with the program as a unique opportunity to enhance trust in the added value of the program.

• Give time for the innovation to spread.

## Competing interests

The authors declare that they have no competing interests.

## Authors' contributions

All authors commented on the paper. HB, AD, PS and SW were involved in the study design. MV conducted the interviews. PS and MV were involved in the data analysis. AD and SW supervised the data collection and analysis. PS wrote the paper with input of all authors. All authors read and approved the final draft.

## Pre-publication history

The pre-publication history for this paper can be accessed here:

http://www.biomedcentral.com/1471-2296/12/94/prepub

## References

[B1] Bodenheimer T Lorig K Holman H Grumbach K Patient self-management of chronic disease in primary care JAMA 2002 288 2469 2475 10.1001/jama.288.19.2469 12435261

[B2] Rijken M Jones M Heijmans M Dixon A Nolte E, McKee M Supporting self-management Caring for people with chronic conditions: a health system perspective 2008 Maidenhead, Open University Press

[B3] National Primary Care Research and Development Centre How has the Expert Patients programme been Delivered and Accepted in the NHS During the Pilot Phase? 2005 Manchester: National Primary care Research and Development Centre http://www.npcrdc.ac.uk/Publications/EPP_briefing_paper.pdf

[B4] Jordan JE Osborne RH Chronic disease self-management education programs: challenges ahead MJA 2007 186 84 87 17223770 10.5694/j.1326-5377.2007.tb00807.x

[B5] Lee V Kennedy A Rogers A Implementing and managing self-management skills training within primary care organisations: a national survey of the expert patients programme within its pilot phase Implementation Science 2006 1 6 10.1186/1748-5908-1-6 16722568 PMC1436011

[B6] Sunaert P Bastiaens H Feyen L Snauwaert B Nobels F Wens J Vermeire E Van Royen P De Maeseneer J De Sutter A Willems S Implementation of a program for type 2 diabetes based on the Chronic Care Model in a hospital-centred health care system: "the Belgian experience" BMC Health Services Research 2009 9 152 10.1186/1472-6963-9-152 19698185 PMC2757022

[B7] Funnell MM Brown TL Childs BP Haas LB Hosey GM Jensen B Maryniuk M Peyrot M Piette JD Reader D Siminerio LM Weinger K Weiss MA National Standards for Diabetes Self-Management Education Diabetes Care 2007 30 1630 1637 10.2337/dc07-9923 17526822

[B8] Bastiaens H Involvement, empowerment and self-management education in primary care. How to support people with type 2 diabetes in self-managing their illness? 2010 PhD thesis, Antwerp University, Department of General Practice, Interdisciplinary Healthcare and Geriatrics

[B9] Grol R Wensing M Eccles M Improving Patient Care. The Implementation of Change in Clinical Practice 2004 Butterworth-Heinemann

[B10] Berwick DM Disseminating Innovations in Health Care JAMA 2003 289 1969 1975 10.1001/jama.289.15.1969 12697800

[B11] Pope C Ziebland S Mays N Qualitative research in health care. Analysing qualitative data BMJ 2000 320 114 116 10.1136/bmj.320.7227.114 10625273 PMC1117368

[B12] Greenhalgh T Robert G Macfarlane F Bate P Kyriakidou O Diffusion of Innovations in Service Organizations: Systematic Review and Recommendations The Milbank Quarterly 2004 82 581 629 10.1111/j.0887-378X.2004.00325.x 15595944 PMC2690184

[B13] Grol R Principles of implementation of changein Grol R Wensing M Eccles M Improving Patient Care The implementation of change 2005 41-57 Elsevier Butterworth Heinemann

[B14] Greenhalgh T Stramer K Bratan T Byrne E Mohammad Y Russell J Introduction of shared electronic records: multi-site case study using diffusion of innovation theory BMJ 2008 337 1786 10.1136/bmj.a1786 PMC3269664 18948344

[B15] Blakeman T Macdonald W Bower P Gately C Chew-Graham C A qualitative study of GPs' attitudes to self-management of chronic disease British Journal of General practice 2006 56 407 414 16762121 PMC1839014

[B16] Vanden Bussche P Desmyter F Duchesnes C Massart V Giet D Petermans J Vyncke V Van Den Noortgate N Willems S Geriatric day hospital: opportunity or threat? A qualitative exploratory study of the referral behaviour of Belgian general practitioners BMC Health Services Research 2010 10 202 10.1186/1472-6963-10-202 20619001 PMC2912898

[B17] Francis CF Feyer A Smith BJ Implementing chronic disease self-management in community settings: lessons from Australian demonstration projects Australian Health Review 2007 31 499 509 10.1071/AH070499 17973606

[B18] Harris MF Williams AM Dennis SM Zwar NA Davies GP Chronic disease self-management: implementation with and within Australian general practice MJA 2008 189 S17 20 19143580 10.5694/j.1326-5377.2008.tb02204.x

[B19] Struijs JN van Til JT Baan CA Experimenting with a bundled payment system for diabetes care in the Netherlands. The first tangible effects 2010 National Institute for Public Health and the Environment (RIVM), Bilthoven, Netherlands http://www.rivm.nl/bibliotheek/rapporten/260224002.html

[B20] Bodenheimer T The Science of Spread How Innovations in Care Became the Norm 2007 California HealthCare Foundation http://www.chcf.org/~/media/MEDIA%20LIBRARY%20Files/PDF/T/PDF%20TheScienceOfSpread.pdf

[B21] Gardner KL Dowden M Togn S Bailie R Understanding the uptake of continuous quality improvement in Indigenous primary health care: lessons from a multi-site case study of the Audit and Best Practice for Chronic Disease project Implementation Science 2010 5 21 10.1186/1748-5908-5-21 20226066 PMC2847538

